# Hydrophilic Polymer Embolization—A Scoping Review of the Available Literature with Focus on Organ Involvement and Outcomes

**DOI:** 10.3390/jcm14020433

**Published:** 2025-01-11

**Authors:** Mohammed Abdulrasak, Haydar Kadim, Ali Someili, Mostafa Mohrag

**Affiliations:** 1 Department of Clinical Sciences, Lund University, 22100 Malmo, Sweden; 2 Department of Gastroenterology and Nutrition, Skane University Hospital, 21428 Malmo, Sweden; 3 Department of Medicine, Faculty of Medicine, Jazan University, Jazan 45142, Saudi Arabia

**Keywords:** hydrophilic polymer, embolization, endovascular therapies, complications

## Abstract

**Background**: Hydrophilic polymer embolization (HPE) is a scarcely reported complication associated with endovascular procedures where the hydrophilic coating dislodges and disseminates to more distal vascular beds, leading to ischemic complications. The aim of this study is to assess the clinical outcomes associated with HPE in the literature and try to quantify it in a scoping manner. **Methods**: All reports with regard to HPE in the PubMed database where clinical data were available were included. Reports were excluded if no clinical data were available and only histopathological descriptions are available, if the language of the report was not in English, and if access could not be obtained to that specific report. **Results**: A total of 60 publications containing 111 patients were identified. The majority (N = 45, 75%) of the publications were “single-patient” case reports. An overwhelming minority of the reports reported underlying hypertension (N = 27, 45.0%) and ischemic heart disease (N = 28, 46.7%). The most common implicated procedures for HPE occurence were cardiac procedures (N = 28, 46.7%), intracranial procedures (N = 13, 21.7%) and aortic procedures (N = 10, 16.7%). Steroids were trialled in nine (15%) of the reports, mainly for HPE to the CNS (7/9), with no mortality in that specific group. However, HPE-related mortality, identified in 48/111 patients, was largely due to HPE with pulmonary and cardiac involvement (combined 36/48 of all deaths). **Conclusions**: HPE seems to be a rare occurrence, although low-quality evidence (mainly case reports) comprises most of the research on the subject. Fatal outcomes seem relatively common, and steroid therapy may be trialled in select cases. Further research, potentially through prospective registry studies may aid in providing more knowledge on HPE.

## 1. Introduction

The past few decades have seen a reduced utilisation of “open” techniques for the treatment of different forms of vascular disease, such as aneurysms and stenosis in various vascular beds [1]. This has been due to a shift towards minimally invasive endovascular methods, often involving the insertion of stents which are deployed inside blood vessels and thus—depending on the underlying pathology—can help increase blood flow by widening the vessel lumen (in case of vascular stenosis) or reduce pressure inside the aneurysm in the case of aneurysmatic diseases, lowering the risk for aneurysmatic rupture. These interventions have, generally speaking, led to a reduced burden of in-patient healthcare, including shortened admission times in comparison to previously more invasive interventions [2]. However, these procedures have their own spectrum of complications as there are procedural risks including local bleeding and dissection or distal embolization of, for example, cholesterol embolization [3].

**Figure 1:**
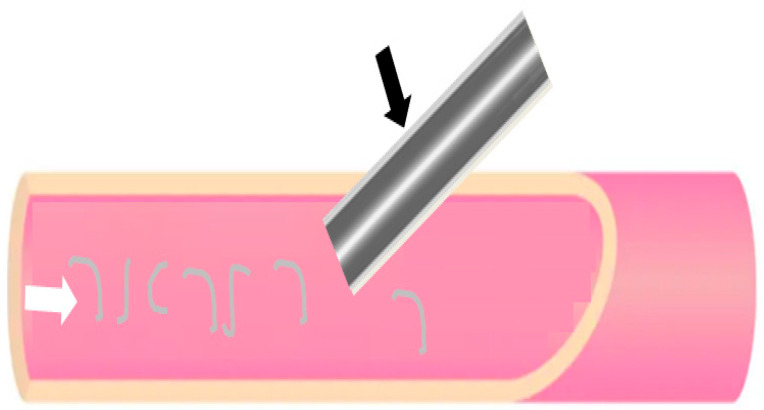
Illustration (not to scale) of an endovascular device (e.g., sheath) being introduced into an artery. The light grey layer (black arrow) on the endovascular device illustrates the hydrophilic polymer coating while the grey lines in the blood vessel illustrates the hydrophilic polymer dissociating from the sheath (white arrow), with potential for distal embolization.

An underreported complication of endovascular procedures is hydrophilic polymer embolization (HPE). This involves embolization of material that covers the surface of endovascular devices, such as stents and the catheter sheath (Figure 1), which can then—through their course in the vessels—move to different organs. This material is, over time, biodegradable [4]. The polymer material can, in the case of embolization, cause organ damage through vascular occlusion; for example, ischemic stroke (in the case of embolization to the central nervous system, CNS), cutaneous ulceration (in the case of embolization to skin vessels), and renal failure (in the case of embolization to the kidneys) amongst other potential adverse events when HPE occurs [4]. Deaths have been reported in association to HPE occurrence in large autopsy series [5].

In this study, the aim is to assess the occurrence of HPE through a review of available reports in the medical literature, with a special focus on the procedures leading to HPE and organs affected by the condition. A secondary aim was to see what kind of literature is available on the condition and what type of further research can be undertaken.

## 2. Materials and Methods

Given the rarity of the condition, a literature review through the PubMed database was undertaken to find the relevant reports. The term “Hydrophilic polymer embolism” was entered in the database. The search was conducted in a semi-systematic (scoping) manner [6], mainly due to the relative rarity of the condition. The abstracts available were screened for suitability of inclusion. All reports where clinical data could be derived were included in the analysis. Reports were excluded if they only entailed pathological descriptions without available clinical data. In addition, reports not in the English language were excluded. Duplicates were excluded when identified. If any additional papers relevant for the purpose of the study were found through a survey of references obtained through PubMed, these were also included. This decision was taken given that the earliest reports may have not contained the current name of the condition (i.e., hydrophilic polymer embolization). All authors participated in the literature review whereby inclusion of the reports was unanimously agreed upon by all authors.

Pertinent data that were included through the review include the patients’ age at intervention leading to HPE, sex, underlying medical illnesses, type of procedure involved, and the clinical outcome. In the cases of multiple procedures being involved, the one with the closest anatomical location to the involved organ was included. The procedures were simplified to entail the organ at which they were aimed and not a specific procedure. For example, percutaneous coronary stenting was classified as cardiac procedure and endovascular coiling of intracranial aneurysms was classified as intracranial procedure. This was implemented due to the wide array of interventions involved in the included patients. In cases where multiple organ involvement of HPE was evident, the organ with the highest “HPE burden” or that leading to death was registered as the main organ. Given the heterogeneity of the reports included, the clinical outcomes were dichotomised to either fatality related to HPE or non-fatal outcome. In cases where any treatment of the condition was attempted, the type of treatment was registered. The pathological details were noted yet not included in the study, given the clinical orientation of the study with a main focus on clinical outcomes.

Data collection was performed through a pre-specified clinical research form. We reasoned that a quantitative approach—albeit the review not adhering to a systematic pattern—was still relevant given the rarity of the condition, and therefore aimed to present the data both in numerical form as percentage of total patients identified and as percentage of total reports. A normal distribution of data was not assumed. Frequencies with percentages and median (Interquartile range, IQR) were presented where applicable. Data analysis was performed using SPSS Statistics 25 software (IBM Corp, Armonk, NY, USA). All numerical data denotes the data available in the case reports and case series included in the analysis. Given that the majority of the data obtained was in the form discrete data for the collected data, such as “pre-HPE” related characteristics (e.g., presence of hypertension), calculating weighted proportions or means was not possible.

## 3. Results

### 3.1. General Characteristics and Types of Procedures Involved

**Figure 2:**
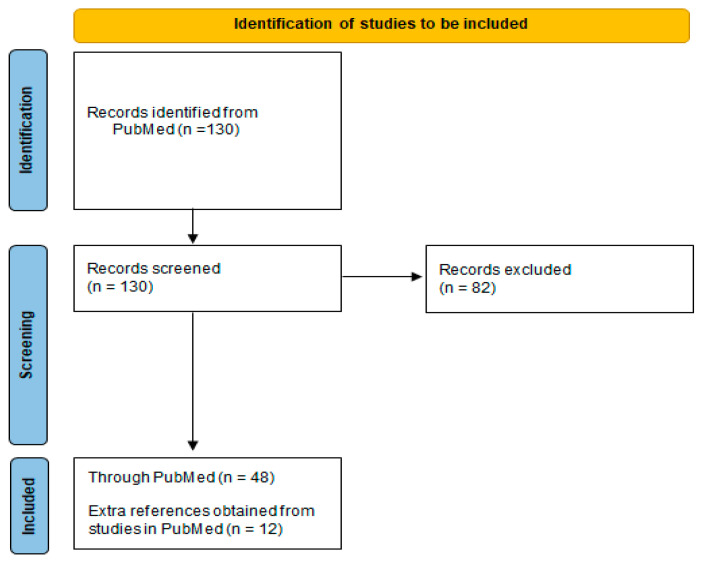
Flow diagram of the included studies in the paper.

Overall, a total of 60 publications were identified as fitting the aforementioned inclusion criteria, all of which were either “single-patient” case reports (N = 45, 75%) or case series’ with ≥2 cases reported (N = 15, 25%) [7,8,9,10,11,12,13,14,15,16,17,18,19,20,21,22,23,24,25,26,27,28,29,30,31,32,33,34,35,36,37,38,39,40,41,42,43,44,45,46,47,48,49,50,51,52,53,54,55,56,57,58,59,60,61,62,63,64,65,66]. Figure 2 shows a flow diagram of the included papers. The majority of the publications included (N = 48, 80% of included) were identified through the PubMed database, while the remaining 12 (20% of included) were identified through the literature review of the references obtained through a PubMed search.

The earliest report of HPE [7] included in the analysis was published in 1997. A total of 111 unique patients were identified, with each publication reporting a median of one (1–2) patient. The largest cases series [30] included in the analysis had 18 (16.2% of total) patients. All publications included in the analysis with relative case contribution per publication are presented in Table S1 of the Supplementary Materials.

The majority (N = 60, 54.1%) of those included were males, aged 65 (54–74) years. The major comorbidities in these patients included hypertension (N = 46, 41.4%), ischemic heart disease (N = 37, 33.3%), peripheral arterial disease including aneurysmatic disease (N = 31, 27.9%), and chronic kidney disease (N = 20, 18.0%). A minority of the patients had underlying malignancy (N = 8, 7.2%). Roughly 8% (N = 9) of patients had diagnoses which did not fit into the pre-defined categories. Detailed patient characteristics are presented in Table 1.

Prior to HPE being diagnosed, a sizeable minority (N = 33, 29.7%) of patients underwent a cardiac procedure, while 24 (21.6%) had a catheter insertion (e.g., central venous catheter or dialysis access), and 23 (20.7%) had an intracranial procedure performed prior to HPE occurrence. Table 2 details the procedure which occurred in the patients.

### 3.2. Organ Involvement and Outcomes

The organ most commonly reported to be involved in HPE was the skin (N = 33, 29.7%). Lungs were involved in 27 (24.3%) cases, the brain in 26 (23.4%) cases, and the heart in 13 (11.7%) cases. Access sites were involved in seven (6.3%) cases and the kidneys in five (4.5%) cases. The diagnosis was established on the basis of biopsy of the involved organ in 62 (55.9%) cases, while post-mortem autopsy with histological examination was the diagnostic test in 44 (39.6%) cases. In five (4.5%) cases, no histological survey was undertaken, yet clinical and/or imaging findings were interpreted as consistent with HPE.

Ten patients (9.1% of patients) in nine reports (15% of reports) received empiric steroid therapy [14,34,44,48,50,61,63,65,66]. These patients had brain involvement (7/10), access site (1/10), and skin involvement (2/10) of HPE. Of these, 6/10 patients had biopsy-confirmed HPE while 4/10 had no biopsy confirmation of HPE.

Fatal outcomes occurred in 48 (43.2%) patients, while 63 (56.8%) patients had non-fatal outcomes. Fatal outcomes were most commonly reported in patients with lung involvement (N = 25, 92.6% of all those with lung involvement). Non-fatal outcomes were most commonly reported in patients with skin involvement (N = 31, 93.9% of all those with skin involvement). HPE involvement in the brain entailed an overwhelmingly non-fatal outcome (N = 17, 65.4% of all those with brain involvement). Table 3 illustrates these details for all groups. Of note, all patients (10/10) who had received steroids had non-fatal outcomes.

## 4. Discussion

In this study, we tried to identify—to the best of our knowledge and by using the most comprehensive medical research database available—all cases where HPE has been reported in the literature and where clinical data could be acquired. We identified 111 unique cases in the literature in 60 publications. This number is not as large as expected initially, especially given the high (and increasing) rate of endovascular procedures performed on a yearly basis. For example, there are over 1 million coronary angiograms [67] performed on a yearly basis in the United States alone. However, there is a likely under-reporting of this condition in the medical literature, based on the potentially elusive nature of establishing diagnosis and the lack of awareness amongst practitioners towards the diagnosis.

Based on the data extracted from the included publications, the patients who seem to suffer from HPE had multiple comorbid conditions, especially cardiovascular ones. This is an expected finding given that the majority of the interventions performed were cardio- or neurovascular procedures. However, a significant minority of patients had HPE diagnosis in association with CVC placement or other catheter insertions, procedures which are generally low risk [68]. This is still a small number when considering the total number of CVCs inserted per year, estimated at 5 million per year [68] in the Unites States alone.

Interestingly, the most commonly involved organ in HPE in our analysis was the skin. This, however, was not unexpected, given the array of dermatological manifestations that HPE to the skin produces, which may mimic different inflammatory conditions [22,57] involving the skin, whereby a biopsy is usually straightforward to perform. Such specific involvement seemed to be generally non-fatal. In spite of this, amputations and loss of limb was reported [35] in association with HPE involvement of the lower limbs and should therefore not be considered an entirely benign entity.

The most fatal involvement of HPE seems to be in the lungs and the heart, with approximately 93% and 85% of each respective involvement being associated with fatal outcomes. Despite this, such a result may have been obtained due to a large proportion of such HPE involvement being identified in patients who had already passed away and undergone an autopsy, resulting in selection bias [16,30,40]. This is especially true given that the autopsy rates in multiple populations have been steadily decreasing [69] and lie at around or just below 5% [70,71], potentially meaning that a large number of “asymptomatic” HPE may be missed. In addition to this, the presence of hydrophilic polymer in the involved organs does not necessarily mean that the cause of death was directly attributed to HPE, as the material is biodegradable [38], and it may be such that the HPE presence was merely due to embolization from a more proximal source. The biodegradability of hydrophilic polymers is, in effect, an Achilles heel with regard to studying the pathogenesis of HPE, given that the actual hydrophilic polymer “load” in each organ may be underestimated due to natural degradation of the polymer substance [62].

Interestingly, steroid therapy was trialled in about 9% of the cases [14,34,44,48,50,61,63,65,66] without any fatal outcomes occurring. This treatment was mainly attempted when HPE to CNS had occurred. The rationale behind treating cerebral HPE with steroids may stem from the histopathological appearance, whereby HPE produces a significant foreign body reaction involving granuloma formation and an accentuated inflammatory response [34]. In addition, the aforementioned publications suggest a resolution of the symptoms associated with cerebral HPE when steroids are utilised, which, in effect, may strengthen the notion that although HPE may act through a vasoocclusive effect [5,72], the inflammatory response may be an even more important driver in the pathogenesis of HPE in areas such as the brain, whereby steroid utilisation is justified to avoid long-term debilitating consequences. This correlates directly to the pathogenesis of HPE which has been detailed in previous reports [5], with an initially vasoocclusive effect caused by the polymer material with ensuing foreign-body reaction with a giant-cell response. However, there is a need to address the fact that hydrophilic polymer is, as previously mentioned, a biodegradable material, whereby the effect of “watchful waiting” may yield just as good recovery as treatment with steroids in those patients who had received them.

There are several limitations to this study. First of all, it was a limited scoping review and not a systematic review with a strict adherence to the PRISMA guidelines [73,74]. This format, however, seemed befitting to quickly assess the knowledge available on the subject and to act as a precursor to future studies on HPE. In addition, we believe it is advantageous to have chosen this format, given that it allowed us to focus on obtaining detailed data from each publication and even obtain highly relevant quantitative data from the available literature in spite of the review not employing a strictly systematic-review-based approach, which may bias the interpretation of the results obtained. Another limitation is having access to one single medical research database (i.e., PubMed) and not using other databases, although PubMed is quite comprehensive and mainly includes research relevant to the biomedical field as compared to other databases (e.g., Scopus or Google scholar). Additionally, another potential room for error is the reliance on the reported comorbidities to assess the general patient characteristics, which may underestimate the actual “comorbidity” burden in these patients as compared to proper chart review, as some of the included case reports included very limited background information on each individual reported case.

We did not undertake any formal “bias risk assessment” [75], mainly because the available evidence constituted solely case reports and case series. Given the aforementioned, the main bias types which may affect our results include publishing bias, given that the cases involving HPE are unique such that journals choose to publish these cases, alongside selection bias, given that cases where HPE may have occurred are more interesting to report with regard to specific outcomes as compared to those with no HPE. In addition, interpretation bias is an issue especially in cases which, e.g., patients received a treatment and the treatment effects may have been interpreted as successful in spite of the outcome potentially being the same through watchful waiting.

To assess the clinical outcomes associated with HPE further, there needs to be multi-centre cooperation to gather—over a longitudinal period of time—a representative number of cases to aid in constructing a case–control study or a retrospective cohort study. Another potential approach to study HPE is to gather data from prospective registries [76], where data are inserted into a registry after performing certain procedures, e.g., endovascular aortic repair or coronary angiography, and—over time—analysing complications that may arise in association with these different procedures. Such research methods are necessary given that the occurrence of HPE is relatively rare and therefore difficult to study in a single-centre fashion. A prospective study design—potentially using registry data—will hopefully be able to ameliorate the risk for bias when studying HPE in the future, and hopefully establish high-quality evidence to aid in establishing “reporting standards” for this currently rarely reported condition.

## 5. Conclusions

In conclusion, HPE seems to be a rare occurrence considering the vast number of endovascular interventions performed yearly. The outcomes of HPE are good if skin is involved; however, HPE may lead to fatal outcomes, especially when critical organs are involved such as the brain or the lungs. Steroid therapy may be trialled in select cases, especially if HPE to CNS occurs. Further prospective studies, potentially through registry-based data, may aid in understanding the rare condition that HPE is and provide more evidence basis for the diagnosis and treatment of this condition.

## Figures and Tables

**Table 1:** Underlying medical characteristics in the included patient cohort.

Characteristic	Frequency * (% Patients)	Frequency ** (% Reports)
Male sex	60 (54.1)	32 (53.3)
Hypertension	46 (41.4)	27 (45.0)
Ischemic cardiac disease	37 (33.3)	28 (46.7)
Valvular cardiac disease	11 (9.9)	10 (16.7)
Other cardiac disease	17 (15.3)	12 (20.0)
Peripheral arterial disease, including aneurysms	31 (27.9)	19 (31.7)
Diabetes	16 (14.4)	13 (21.7)
Cerebrovascular disease	10 (9.0)	7 (11.7)
Hepatic disease	4 (3.6)	3 (5.0)
Chronic kidney disease	20 (18.0)	13 (21.7)
Organ transplant recipient	9 (8.1)	6 (10.0)
Inflammatory disorders (e.g., Rheumatoid)	6 (5.4)	6 (10.0)
Underlying malignancy	8 (7.2)	5 (8.3)
Blood disorder (e.g., sickle-cell anaemia)	3 (2.7)	3 (5.0)
Others	9 (8.1)	6 (10.0)

* N_1_ = 111 patients; ** N_2_ = 60 reports.

**Table 2:** Procedures which the included patients underwent.

Type of Procedure	Frequency * (% Patients)	Frequency ** (% Reports)
Intracranial procedure	23 (20.7)	13 (21.7)
Cardiac procedure	33 (29.7)	28 (46.7)
Aortic procedure	17 (15.3)	10 (16.7)
Peripheral arterial intervention	8 (7.2)	8 (13.3)
Catheter insertion	24 (21.6)	9 (15.0)
Complex procedure, e.g., organ transplant	6 (5.4)	3 (5.0)

Frequency (% reports) contributed per report in numerical form as percentage of total patients identified and as percentage of * N_1_ = 111 patients: ** N_2_ = 60 reports.

**Table 3:** Patients stratified by fatal versus non-fatal outcomes.

Organ Involved	Non-Fatal Outcome (N = 63)	Fatal Outcome (N = 48)
Brain	17	9
Access site	6	1
Lung	2	25
Skin	31	2
Heart	2	11
Kidney	5	0

## Data Availability

The data supporting the findings of this study are available from the corresponding author upon reasonable request.

## Supplementary Materials

The following supporting information can be downloaded at: https://www.mdpi.com/article/10.3390/jcm14020433/s1, Table S1: included reports with year published and “case contribution” per study; Table S2: PRISMA checklist adapted from [77].
