# Supplementary material for: Hydrophilic Polymer Embolization—A Scoping Review of the Available Literature with Focus on Organ Involvement and Outcomes
Source: J Clin Med. 2025 Jan 11;14(2):433. doi: 10.3390/jcm14020433 (PMC11765792; doi:10.3390/jcm14020433)
Supplement: Supplementary file 1 [file jcm-14-00433-s001.zip › jcm-3399610-supplementary.pdf]

| *Name of publication with hyperlink* | *Year* | *Cases contributed* |
|---|---|---|
| Foreign Bodies in Small Arteries after Use of an Infusion Microcatheter | 1997 | 4 |
| Nonhealing Wound Resulting From a Foreign-Body Reaction to a Radial Arterial Sheath | 2003 | 1 |
| Sterile inflammation after transradial catheterization using a hydrophilic sheath | 2005 | 1 |
| Complications of Endovascular Polymers Associated With Vascular Introducer Sheaths and Metallic Coils in 3 Patients, With Literature Review | 2008 | 3 |
| Sterile abscess formation as a complication of hydrophilic radial artery cannulation | 2008 | 1 |
| Embolization of Hydrophilic Catheter Coating to the Lungs | 2009 | 1 |
| Intravascular polymer material after coil embolization of a giant cerebral aneurysm | 2009 | 1 |
| Steroid-responsive sterile inflammation after transradial cardiac catheterisation using a sheath with hydrophilic coating* | 2009 | 1 |
| Hydrophilic polymer emboli: an under-recognized iatrogenic cause of ischemia and infarct | 2010 | 8 |
| Autopsy demonstration of intramyocardial polymer gel emboli associated with a giant-cell reaction following cardiac catheterization: a case report | 2012 | 1 |
| Embolization of hydrophilic coating material to small intracardial arteries after multiple percutaneous transluminal angioplasty procedures | 2012 | 1 |
| Polymer Embolization From Minimally Invasive Interventions | 2013 | 3 |
| Histopathological assessment of fatal ipsilateral intraparenchymal hemorrhages after the treatment of supraclinoid aneurysms with the Pipeline Embolization Device | 2014 | 3 |
| Hydrophilic polymer embolism induced acute transcatheter aortic valve thrombosis: a novel complication | 2014 | 1 |
| Myocardial hydrophilic polymer emboli following cardiac catheterization: a case report and literature review | 2014 | 1 |
| Hydrophilic Polymer Embolization: An Emerging Cause of Livedo Reticularis | 2014 | 1 |
| Hydrophilic polymer microemboli in a patient with a chronic cutaneous ulcer: a case report | 2014 | 1 |
| Hemorrhagic Panniculitis Caused by Delayed Microemboli From Intravascular Device | 2014 | 1 |
| Cerebral foreign body reaction after carotid aneurysm stenting | 2015 | 1 |
| Fatal Case of Hydrophilic Polymer Emboli | 2015 | 1 |
| Acute Hydrophilic-Polymer Nephropathy and Acute Renal Failure | 2015 | 1 |
| Cutaneous microemboli from hydrophilic polymer after endovascular procedures | 2015 | 8 |
| Distal Embolization of Hydrophilic-Coating Material From Coronary Guidewires After Percutaneous Coronary Interventions | 2015 | 4 |
| Hydrophilic polymer embolism and associated vasculopathy of the lung: prevalence in a retrospective autopsy study | 2015 | 18 |
| Intracoronary Embolization of the Coating Polymer on a Cardiovascular Device | 2015 | 1 |
| Hydrophilic polymer embolization: another differential diagnosis of iatrogenic leg embolization after transcatheter aortic valve replacement | 2016 | 1 |
| Hydrophilic polymer microembolism with cutaneous involvement observed incidentally in a patient undergoing endovascular procedures: a case report | 2016 | 1 |
| Cerebral granulomatous inflammation secondary till HPE following thromboectomy* | 2017 | 1 |
| HP vasculopathy with coinciding pseudoxanthoma elasticum like changes in an amputated toe | 2017 | 1 |
| Fatal Sickling Triggered by Massive Foreign Particle Embolism: A Case Report of Unrecognized Indwelling Venous Catheter Drug Abuse in Sickle Cell Disease | 2018 | 1 |
| Cutaneous hydrophilic polymer embolism accompanied by acute renal failure | 2018 | 1 |
| Collapsing Glomerulopathy Associated With Hydrophilic Polymer Emboli | 2018 | 1 |
| Left cerebral hemisphere hydrophilic polymer embolism associated with endovascular WEB treatment of a ruptured aneurysm of the anterior cerebral artery | 2018 | 1 |
| Hydrophilic Polymer Embolism in the Lung as an Adverse Event | 2018 | 1 |
| Hydrophilic polymer embolization after thoracic endovascular aortic repair | 2019 | 2 |
| Cutaneous hydrophilic polymer emboli following thoracic endovascular aortic repair | 2019 | 1 |
| Ischemic Toe Ulceration Due to Foreign Body Embolus From Hydrophilic Polymer-Coated Intravascular Device | 2019 | 1 |
| Cerebral foreign body reaction due to hydrophilic polymer embolization following aneurysm treatment by pipeline flow diversion device* | 2019 | 1 |
| First case report of the coexistence of hydrophilic polymer embolism and cholesterol crystal embolism associated with an endovascular procedure | 2020 | 1 |
| Images in Vascular Medicine: Hydrophilic polymer emboli after percutaneous coronary intervention due to severe tortuosity and a calcified nodule of the aortic access route | 2021 | 1 |
| Foreign body reaction toward hydrophilic polymer at the site of endovascular procedure: A report of two cases | 2021 | 2 |
| Hydrophilic Polymer Embolization Following Coiling of Intracranial Aneurysm* | 2021 | 1 |
| Cutaneous Polymer-Coating Embolism After Endovascular Procedures: Report of Two Cases and a Literature Review | 2021 | 2 |
| Medical treatment of polymeric cerebral granulomatous reactions following endovascular procedures* | 2021 | 3 |
| Late-onset Cutaneous Hydrophilic Polymer Embolism: A Case Occurring Two Years after Endovascular Procedures | 2021 | 1 |
| The diagnostic utility of Elastica van Gieson stain in hydrophilic polymer embolism | 2021 | 1 |
| Hydrophilic polymer embolism identified in brain tumor specimens following Wada testing: A report of 2 cases | 2021 | 2 |
| Hydrophilic polymer embolization following flow diversion of cerebral aneurysms | 2021 | 1 |
| Embolized Hydrophilic Coating Polymers Found in Left Ventricular Assist Device Apical Core Specimen | 2022 | 1 |
| Cutaneous hydrophilic polymer emboli following endovascular repair of an abdominal aortic aneurysm: a case and review of literature | 2022 | 1 |
| Livedo racemosa associated with central venous catheter use in a newborn | 2022 | 1 |
| Polymer Emboli Complicating Transcatheter Aortic Valve Implantation | 2022 | 1 |
| Hydrophilic polymer gel embolus in a kidney transplant biopsy | 2022 | 1 |
| Fragments of hydrophilic polymer coating as an embolic material – a rare complication and potential cause of death | 2023 | 1 |
| A Rare Case of Hydrophilic Polymer Embolization After Transcatheter Aortic Valve Replacement* | 2023 | 1 |
| Cutaneous Hydrophilic Polymer Embolism in a Patient with Acute Kidney Injury Post Endovascular Procedure | 2023 | 1 |
| Cerebral foreign body granulomas after mechanical thrombectomy: Two case reports and a review of the literature* | 2023 | 2 |
| Hydrophilic polymer embolization after intravascular lithotripsy | 2023 | 1 |
| Delayed cerebral complications associated with endovascular catheter coil embolization for pulmonary venous shunts mimicking cerebral inflammatory diseases* | 2024 | 1 |
| Cutaneous Hydrophilic Polymer Embolism: An Important and Overlooked Clinical Entity* | 2024 | 2 |

Supplementary Table S1 – included reports with year published and "case contribution" per study. Hyperlink to PubMed can be obtained through hovering on the specific report. Asterisk (*) denotes report where steroid therapy was trialled

| SECTION | ITEM | PRISMA- CHECKLIST ITEM | REPORTED ON PAGE # |
|---|---|---|---|
| **TITLE** | | | |
| Title | 1 | Identify the report as a scoping review. | 1 |
| **ABSTRACT** | | | |
| Structured summary | 2 | Provide a structured summary that includes (as applicable): background, objectives, eligibility criteria, sources of evidence, charting methods, results, and conclusions that relate to the review questions and objectives. | 1 |
| **INTRODUCTION** | | | |
| Rationale | 3 | Describe the rationale for the review in the context of what is already known. Explain why the review questions/objectives lend themselves to a scoping review approach. | 2-3 |
| Objectives | 4 | Provide an explicit statement of the questions and objectives being addressed with reference to their key elements (e.g., population or participants, concepts, and context) or other relevant key elements used to conceptualize the review questions and/or objectives. | 3 |
| **METHODS** | | | |
| Protocol and registration | 5 | Indicate whether a review protocol exists; state if and where it can be accessed (e.g., a Web address); and if available, provide registration information, including the registration number. | 3 |
| Eligibility criteria | 6 | Specify characteristics of the sources of evidence used as eligibility criteria (e.g., years considered, language, and publication status), and provide a rationale. | 3 |
| Information sources* | 7 | Describe all information sources in the search (e.g., databases with dates of coverage and contact with authors to identify additional sources), as well as the date the most recent search was executed. | 3 |
| Search | 8 | Present the full electronic search strategy for at least 1 database, including any limits used, such that it could be repeated. | 3 |
| Selection of sources of evidence† | 9 | State the process for selecting sources of evidence (i.e., screening and eligibility) included in the scoping review. | 3 |
| Data charting process‡ | 10 | Describe the methods of charting data from the included sources of evidence (e.g., calibrated forms or forms that have been tested by the team before their use, and whether data charting was done independently or in duplicate) and any processes for obtaining and confirming data from investigators. | 2-3 |
| Data items | 11 | List and define all variables for which data were sought and any assumptions and simplifications made. | 2-3 |
| Critical appraisal of individual sources of evidence§ | 12 | If done, provide a rationale for conducting a critical appraisal of included sources of evidence; describe the methods used and how this information was used in any data synthesis (if appropriate). | N.A |
| Synthesis of results | 13 | Describe the methods of handling and summarizing the data that were charted. | 3 |
| **RESULTS** | | | |
| Selection of sources of evidence | 14 | Give numbers of sources of evidence screened, assessed for eligibility, and included in the review, with reasons for exclusions at each stage, ideally using a flow diagram. | 3, appendix |
| Characteristics of sources of evidence | 15 | For each source of evidence, present characteristics for which data were charted and provide the citations. | 3-4, appendix |
| Critical appraisal within sources of evidence | 16 | If done, present data on critical appraisal of included sources of evidence (see item 12). | N.A |
| Results of individual sources of evidence | 17 | For each included source of evidence, present the relevant data that were charted that relate to the review questions and objectives. | 3-5 |
| Synthesis of results | 18 | Summarize and/or present the charting results as they relate to the review questions and objectives. | 3-5 |
| **DISCUSSION** | | | |
| Summary of evidence | 19 | Summarize the main results (including an overview of concepts, themes, and types of evidence available), link to the review | 5-6 |

| | | | |
|---|---|---|---|
| | | **questions and objectives, and consider the relevance to key groups.** | |
| **Limitations** | **20** | **Discuss the limitations of the scoping review process.** | **6** |
| **Conclusions** | **21** | **Provide a general interpretation of the results with respect to the review questions and objectives, as well as potential implications and/or next steps.** | **6-7** |
| **FUNDING** | | | |
| **Funding** | **22** | **Describe sources of funding for the included sources of evidence, as well as sources of funding for the scoping review. Describe the role of the funders of the scoping review.** | **N.A** |

Supplementary Table S2 – PRISMA checklist adapted from "Tricco AC, Lillie E, Zarin W, O'Brien KK, Colquhoun H, Levac D, et al. PRISMA Extension for Scoping Reviews (PRISMAScR): Checklist and Explanation. Ann Intern Med. 2018;169:467–473. doi: 10.7326/M18-0850."
